# Facelift: Assessment of Total Platysma Muscle Transection to Prevent the Recurrence of Platysmal Bands

**DOI:** 10.1007/s00266-023-03664-w

**Published:** 2023-09-22

**Authors:** Jean-Paul Meningaud, Rosita Pensato, Virginie Pineau, Luca D’Andrea, Chiara Pizza, Edoardo Coiante, Barbara Hersant, Simone La Padula

**Affiliations:** 1 Department of Plastic, Reconstructive and Maxillofacial Surgery, Henri Mondor Hospital, University Paris XII, 1 rue Gustave Eiffel, 94000 Créteil, France; 2 Department of Plastic and Reconstructive Surgery, Università degli studi di Napoli Federico II, Via Pansini 5, 80131 Naples, Italy; 3 Paris, France

**Keywords:** Facelift, Rejuvenation surgery, SMAS flap, Platysma muscles transection, Platysma myotomy, The Face- and Neck-Lift Objective Photo-Numerical Assessment Scale

## Abstract

**Introduction:**

Determining which facelift technique yields the most effective long-term rejuvenation results and ensures optimal stability over time remains a significant question in cosmetic surgery: Does the most invasive surgery lead to the best long-term outcomes? This study aims to evaluate the authors’ approach using total platysma muscle transection to prevent platysma band recurrence, and to provide anatomical observations supporting and justifying their procedure.

**Material and Methods:**

A preliminary study in anatomical basic sciences was conducted to establish the rationale for our method. A prospective single-blind study was conducted, involving eighty patients seeking facial rejuvenation with platysmal band correction. They underwent face and neck-lift procedures with total platysma transection by the same surgeon between May 2013 and May 2016. Cosmetic outcomes were assessed using the Face and Neck-Lift Objective Photo-Numerical Assessment Scale. Scores by three blind evaluators before surgery, at 1 and 5 years postoperatively, were compared using a matched *T* Test (*p* < 0.05).

**Results:**

The preliminary anatomical study revealed a consistent anastomotic system between the cervical branch of the facial nerve and the branches of the cervical plexus. Incomplete platysma section during a facelift might contribute to platysma band recurrence. The clinical study demonstrated satisfactory outcomes, with significant overall appearance improvement (*p* < 0.00001) and no platysma band recurrence. Complication rate was low.

**Conclusion:**

The authors’ technique achieved satisfactory long-term results with minimal complications. However, due to the lengthy operating time and steep learning curve, it should be reserved for highly motivated patients.

**Level of Evidence II:**

This journal requires that authors assign a level of evidence to each article. For a full description of these Evidence-Based Medicine ratings, please refer to the Table of Contents or the online Instructions to Authors www.springer.com/00266.

**Supplementary Information:**

The online version contains supplementary material available at 10.1007/s00266-023-03664-w.

## Introduction

The facial aging process is characterized by an important loss of skin elasticity, an increased resting tone of the facial and neck muscles [1], and a downward migration of adipose and glandular tissues [2]. Clinically, this process may cause sagging cheeks, a loss of the oval shape of the face, a double chin, and a downward migration of the labial commissures [3]. The appearance of cervical wrinkles and platysmal bands, mainly observed in the paramedian and posterolateral cervical area, is also frequent. Reliable evaluation criteria for cervical rejuvenation procedures were described in 1980 by Ellebogen [4] and remained virtually unchanged since. Surgical treatment of this anatomical region should aim at restoring the following criteria: a cervicofacial angle of 105–120°, a sub-hyoid depression, a prominent inferior mandibular border, a visible thyroid cartilage bulge, and a visible anterior sternocleidomastoid border. Several techniques for the treatment of platysma bands have been described since the early 1970s, initially by Skoog [5] and then by Guerrerosantos [6, 7], Cornell [8, 9] and Aston [10]. Optimal treatment of platysmal bands is still controversial [1, 2, 11–14]. Transection of the platysma muscles appears to be a good strategy for the management of cervicofacial aging in severe cases to avoid recurrence. The cervical lift technique should always be performed simultaneously with the facelift, considering the continuity of facial and cervical anatomical structures [1–14]. The objective of this study is to evaluate the authors’ approach, which involves utilizing the total platysma muscle transection technique, in order to prevent the recurrence of platysmal bands, and to provide anatomical observations supporting and justifying their procedure.

## Material and Methods

**Fig. 1:**
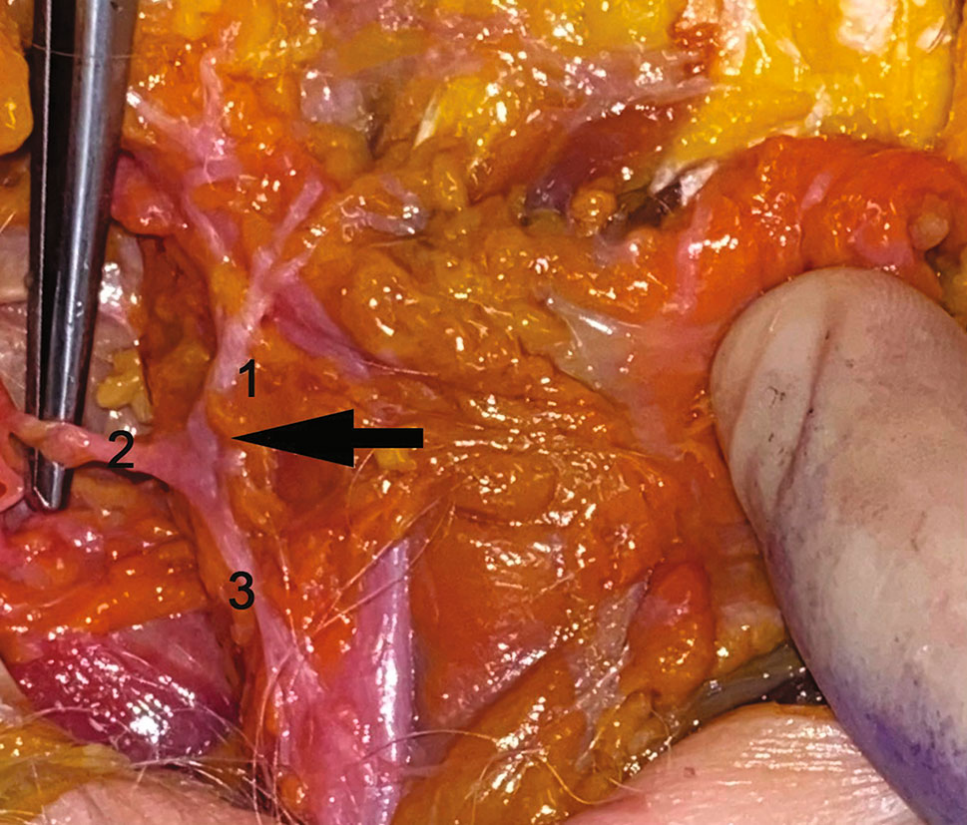
Anatomical findings in fresh cadaver dissections. Ten consecutive cadaveric face and neck dissection (20 hemi-necks and faces) of the superficial musculo-aponeurotic system (SMAS) and platysma areas were carried out to study the innervation of the platysma muscle. Dissections were performed using a facelift approach. The skin and the SMAS were raised very cautiously to reveal the underlying nerves. Innervation of the platysma muscle is shown: The platysma muscle is innervated by the cervical branch of the facial nerve that is connected to the nerves arising from the cervical plexus. A constant anastomotic system between the cervical branch of the facial nerve and the branches of the cervical plexus may be responsible of the platysmal bands recurrence after a facelift procedure, when a complete section of the platysma is not carried out. 1. Marginal mandibular branch of the facial nerve. 2. Cervical branches of the facial nerve. 3. Branches of the cervical plexus. Arrow: Anastomoses between the cervical branch of the facial nerve and branches of the cervical plexus

A preliminary study in anatomical basic sciences was conducted before the initiation of the clinical study to demonstrate the rationale behind our method. We performed ten consecutive cadaveric face and neck dissections (20 hemi-necks and faces) of the superficial musculo-aponeurotic system (SMAS) and platysma areas to study the innervation of the platysma muscle. The dissections were performed using a facelift approach, with utmost care in raising the skin, SMAS, and platysma to reveal the underlying nerves. High-resolution photographs were taken and collected (Fig. 1). The authors subsequently carried out a prospective single-blind study on patients seeking facial rejuvenation surgery with correction of platysmal bands, describing the total platysma transection technique. In our practice, neck rejuvenating surgery, using a neck-lift with total platysma transection, was consistently combined with a facelift. Patients who presented to our center between May 2013 and May 2016, seeking a face and neck-lift for the correction of platysmal bands, were enrolled consecutively in this study. The study’s exclusion criteria included individuals who had undergone previous rejuvenation surgery, received botulinum toxin injections within the last 6 months, hyaluronic acid injections or laser treatments within the past year. Other exclusion criteria encompassed individuals with uncontrolled diabetes (determined through glycated hemoglobin examination), obese individuals (with a BMI equal to or exceeding 30), and patients with cognitive deficits impeding comprehension and consent. Patients who were smokers were advised to stop smoking for at least one month before and one month after the surgery. To ensure compliance, all patients with a history of smoking were tested 15 days before and the day before the surgery using Screen Pharma Check, a rapid test for the qualitative detection of cotinine (a nicotine metabolite) in human urine. If they tested positive, they were excluded from the study. Additionally, patients with a previous smoking history were monitored after surgery to assess their abstinence from smoking. Qualitative detection of cotinine in human urine was repeated in these patients 15 and 30 days after the procedure. If they tested positive, they were excluded from the analysis. Although a history of smoking was not an exclusion criterion, patients who did not cease smoking at least one month before the surgery and were non-compliant after the procedure were excluded from the study. All eligible patients were informed of the risks and benefits of the proposed procedure, received detailed information about the study, and provided written informed consent to participate. The procedures performed in the study were in concordance with the ethical standards of the institutional and/or national research committee and the 1964 Declaration of Helsinki and its subsequent amendments or comparable ethical standards. To evaluate the effectiveness of the authors’ method, we utilized the Face and Neck-Lift Objective Photo-Numerical Assessment Scale (Table 1), which was developed by our team. Three raters were involved in the assessment process [15, 16]. Preoperative photographs of the patients were taken, as well as photographs at 1 and 5 years postoperatively. Each photograph was anonymized and assigned to a separate scorecard. These scorecards were then independently evaluated by three blinded assessors, including a plastic surgeon, a maxillofacial surgeon, and a dermatologist. Evaluators were instructed to use our scale to assess the preoperative and postoperative (1- and 5-year postoperative) photographs. They were unaware of both the ages and the specific procedure performed. To assess the severity of the aging signs, mid face, lower face and neck scales were used. In each scale, the severity degree ranged from 0 (no sign) to 3 (very intense or visible signs). The final score for one patient ranged from 0 to 33. The evaluators were asked to use four items (0–3) and to note the signs of aging severity for each esthetic region (mid face, lower face and neck). Scores obtained preoperatively 1 and 5 years postoperatively were compared using a matched *T* test. A value of *p* < 0.05 was considered significant. Descriptive statistics were used to present patient characteristics. The statistical analysis was conducted using STATA version 17 (StataCorp LLC, USA). All authors had full access to the database and took full responsibility for its integrity.

**Table 1:** The Face- and Neck-Lift Objective Photo-Numerical Assessment Scale

*Mid face*				
Cheek fullness	Full cheek	Mildly sunken cheek	Moderately sunken cheek	Severely sunken cheek
	0	1	2	3
Cheek ptosis	No ptosis	Mild ptosis	Moderate ptosis	Severe ptosis
	0	1	2	3
Lower face jawline	No sagging	Mild sagging	Moderate sagging	Severe Sagging
	0	1	2	3
Nasolabial folds	No folds	Mild folds	Moderate folds	Severe folds
	0	1	2	3
Marionette lines	No lines	Mild lines	Moderate lines	Severe lines
	0	1	2	3
Perioral wrinkles	No wrinkles	Mild wrinkles	Moderate wrinkles	Severe wrinkles
	0	1	2	3
Oral commissures	No downturn	Mild downturn	Moderate downturn	Severe downturn
	0	1	2	3
*Neck*				
Neck folds	No folds	Mild folds	Moderate folds	Severe folds
	0	1	2	3
Double chin	No double chin	Mild double chin	Moderate double chin	Severe double chin
	0	1	2	3
Platysmal bands	No platysmal bands	Mild platysmal bands	Moderate platysmal bands	Severe platysmal bands
	0	1	2	3
Submandibular glands ptosis	No ptosis	Mild ptosis	Moderate ptosis	Severe ptosis
	0	1	2	3

### Neck Rejuvenation Surgery

The surgery was performed under general anesthesia. To achieve complete facial exposure and avoid accidental extubation, an armored orotracheal tube attached to the palatal aspect of a maxillary premolar with a 4/0 wire was used for all patients. One hundred milliliters (ml) of tumescent solution—1 mg of adrenaline in 1000 ml of normal saline solution (0.9% NaCl)—were injected on each side through the incision lines and into the dissection area above the superficial musculo-aponeurotic system (SMAS) plane using a 1-mm diameter cannula. As we operate on general anesthesia, we prefer not to use local anesthetic that may prevent to monitor the facial nerve (motion if cautery close to the nerve) and because lidocaine, for example, is a vasodilatator that may increase the bleeding. Recently, we added tranexamic acid.

### Submental Lipectomy

Submental lipectomy (through a horizontal medial submental incision 4-cm-long (1.57 inch), located 1 cm (0.39 inches) posterior to the mento-cervical fold, was performed using Metzenbaum scissors under direct vision, preserving a thin layer of subcutaneous fat. If necessary, the lipectomy was completed underneath the platysma muscle, and especially between the anterior bellies of the digastric muscles. Only in case of important fatty tissue excess, this phase was preceded by a liposuction.

### Full Section of the Platysma Muscle and Platysma Suture

**Fig. 2:**
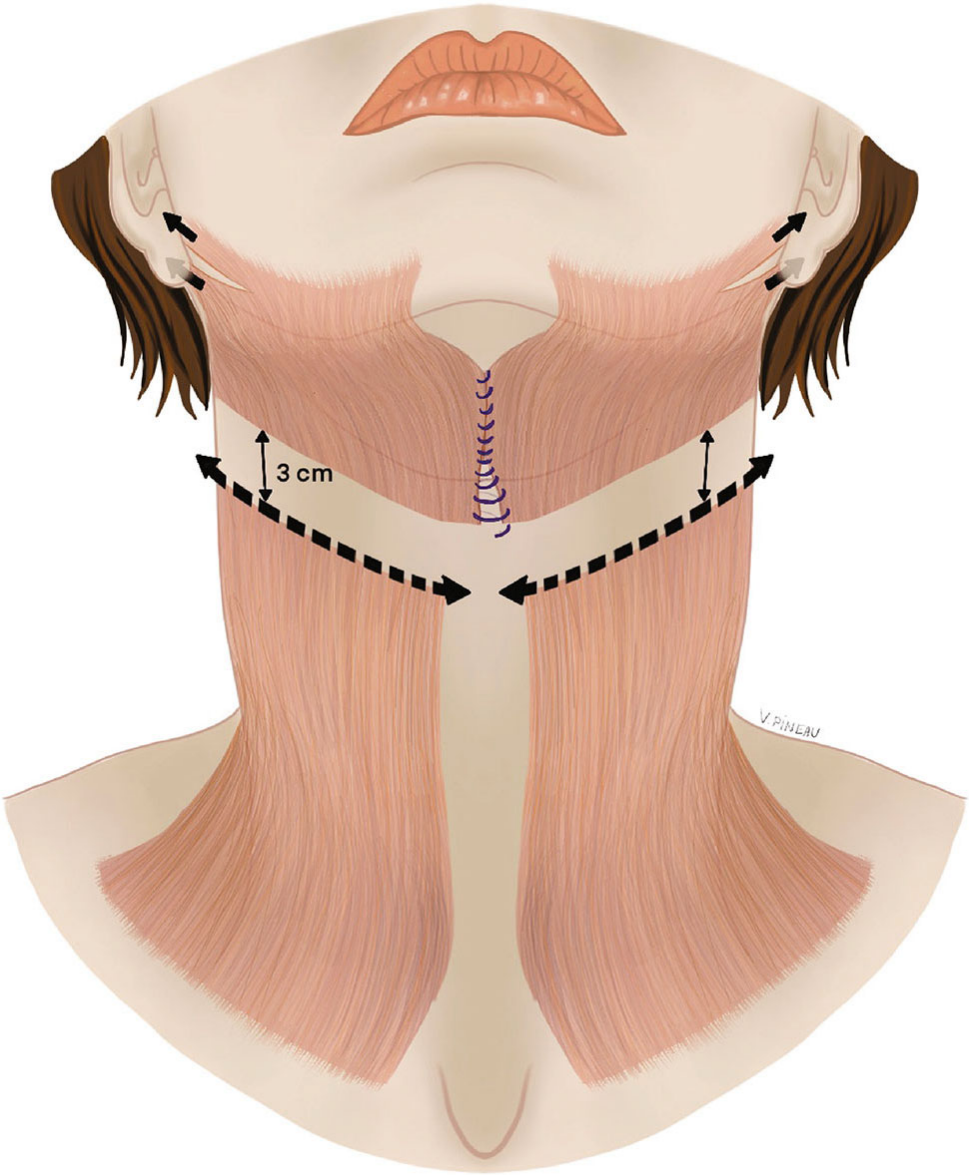
Total platysmal transection. Three cm (1.2 inches) were maintained between the edges of the transected platysma to avoid the recurrence of the platysmal bands

The cervical skin was completely dissected and raised (from side to side) up to a line running at least 3 cm (1.2 inches) below the upper border of the thyroid cartilage. Anterior and posterior edges of platysma were identified, then the muscle was undermined from one edge to the other starting from the inferior mandibular border and extending downwards to a point located at least 3 cm below the upper border of the thyroid cartilage. This was followed by its complete transection. Myotomy was performed to prevent any reattachment. Three cm (1.2 inches) were maintained between the edges of the transected platysma to avoid the recurrence of the platysmal bands (Fig. 2). All the procedure was performed with 3X led magnification glasses to check out that no platysma fibers remained attached. The muscle section was performed through two approaches, using the submental incision for the medial segment and the peri-auricular approach for the lateral segment. After the platysma transection, the submandibular glands were identified. The suture of the anterior bellies of the digastric muscles with 2/0 PDS® (Ethicon®) stiches facilitated the identification of the submandibular glands by bringing them to the midline. In case of significant ptosis or hypertrophy of the submandibular glands, partial resection was performed to improve the cervical contours [17]. To avoid bleeding complications, the glandular section was performed using a LigaSure® device (Covidien®). It is a sealing device that uses the body’s own collagen and elastin to create a permanent fusion zone. Fifteen UI botulinum toxin (Vistabel®, Allergan®) was immediately injected into the residual glands. Such injections have been shown to reduce the incidence of posttraumatic sialocele. [18, 19]. The glandular capsules were closed with a 3/0 Vicryl® suture. A suture of the medial edges of the platysma was then performed. An edge-to-edge anterior running suture with a 3/0 PDS® (Ethicon®) was performed, from the chin to the upper border of the thyroid cartilage. The platysma was then attached through an anchoring suture to the hyoid bone (Video 1). Careful hemostasis was performed.

### Facelift

**Fig. 3:**
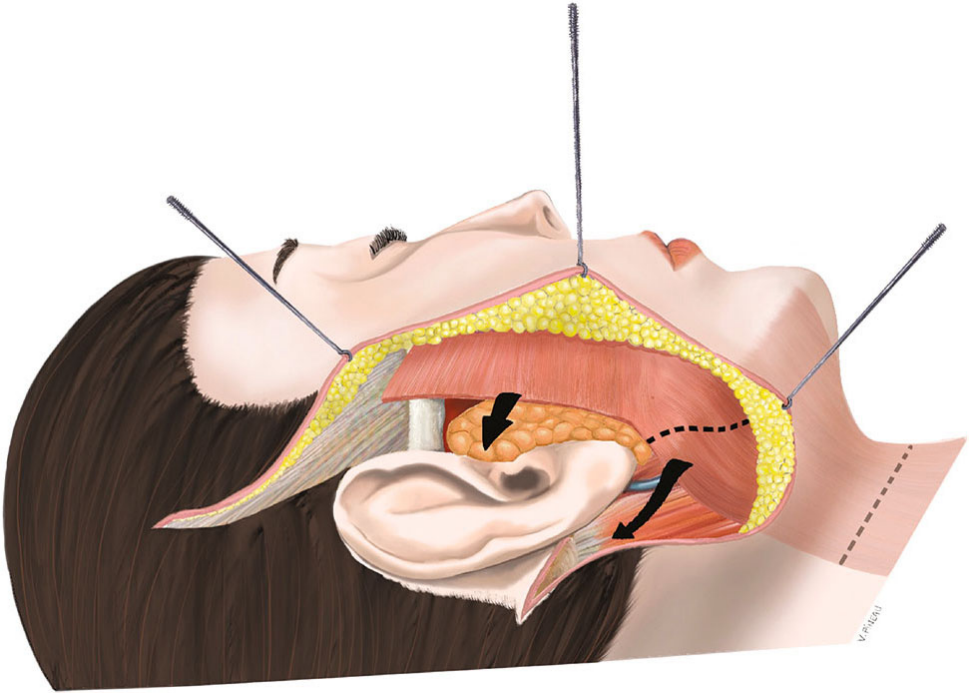
Face and neck-lift. A SMAS flap is raised and dissected above the entire parotid region starting from the pre-auricular incision. The SMAS and platysma muscle are raised after making a 2 cm (0.79 inches) incision below the earlobe to allow traction with a horizontal vector and a vertical vector. The lower head of the platysma muscle is then pulled following a retro-mastoidal horizontal vector and anchored to the mastoidal aponeurosis, and the superior head is pulled on a vertical vector and anchored to the pre-auricular aponeurosis through Vicryl® 2/0 sutures

The peri-auricular approach was designed bilaterally: a vertical line descending in front of the anterior edge of the ear and then behind the tragus [14, 20], running around the earlobe, into the retro-auricular groove to the mastoid prominence and descending into the scalp toward the nape. Skin dissection was performed using Trepsat’s spatulated-tipped dissection scissors (Pouret Medical®, Clichy, France), initially in a strict subcutaneous plane, above the SMAS up to the commissural region. Then, the dissection of the cervical skin flap was performed in the same plane from the retro-auricular incision to the medial region. Afterward, the SMAS flap was raised and dissected above the entire parotid region starting from the pre-auricular incision. The SMAS and platysma flap were raised after doing a 2 cm (0.79 inches) incision below the earlobe to allow traction with a horizontal vector and a vertical vector (Fig. 3). The lower strip was pulled following a retro-auricular horizontal vector and anchored to the mastoid fascia, and the superior strip was pulled following a vertical vector and anchored to the temporal fascia with PDS® 2/0 sutures (Ethicon®). The SMAS and platysma dissection had to be effective enough to redefine the mandible (inferior border and angle) and the submental contours. Before anchoring the flaps, care was taken to avoid damaging the great auricular nerve and the external jugular vein while repositioning the platysma. In the case of parotidomegaly, a PDS® 3/0 (Ethicon®) running suture on the parotid fascia was carried out. Careful and accurate hemostasis was performed, and the pre-auricular skin was lifted backwards and upwards. The undermined skin was incised below the earlobe to obtain a superior and posterior skin flap on either side of the earlobe which followed the same vectors used for the SMAS and the platysma. Then, a resection of the redundant skin was carefully performed to achieve a precise juxtaposition of the skin edges. The skin closure was performed with buried Monocryl® 4/0 (Ethicon®) stiches and Vicryl Rapid® 5/0 (Ethicon®) intradermal running suture. Suction drains were used at the time of the study.

When patients expressed a desire to improve the appearance of perioral rhytides and enhance the skin quality in the malar and periocular regions, nanofat injection was incorporated into the facelift procedure. Nanofat, derived from autologous fat collection, was acquired using a small-gauge cannula measuring 1 mm in diameter, attached to a 10-ml Luer lock syringe. The collected fat underwent subsequent refinement using a Tulip kit. This refining procedure entailed the passage of the adipose graft through a series of three reducers (with diameters of 2.4, 1.4, and 1.2 mm), followed by traversal through a nano-filter. When requested, upper and lower blepharoplasty was also performed at the end of the cervicofacial lift. All the procedures were performed by the same surgeon.

### Postoperative Care

A refrigerant system (Hilotherm®, Hilotherapy® UK Ltd, Coventry) was applied to the treated areas immediately after surgery. Patients were instructed to wear a compressive dressing and to apply vitamin A ointment daily on the perioral region for 7 days. To reduce pain, patients were routinely given paracetamol and corticosteroids. Daily massages of the scars and of the cervical region were recommended for 3 months, starting 3 weeks after surgery.

## Results

The 20 hemi-neck and face dissections consistently revealed a rich and constant anastomotic system between the cervical branch of the facial nerve and the branches of the cervical plexus (Fig. 1). In our view, these connections could potentially contribute to the frequent recurrence of platysma bands after a facelift procedure, particularly when a complete section of the platysma, as previously described, is not performed.

**Fig. 4:**
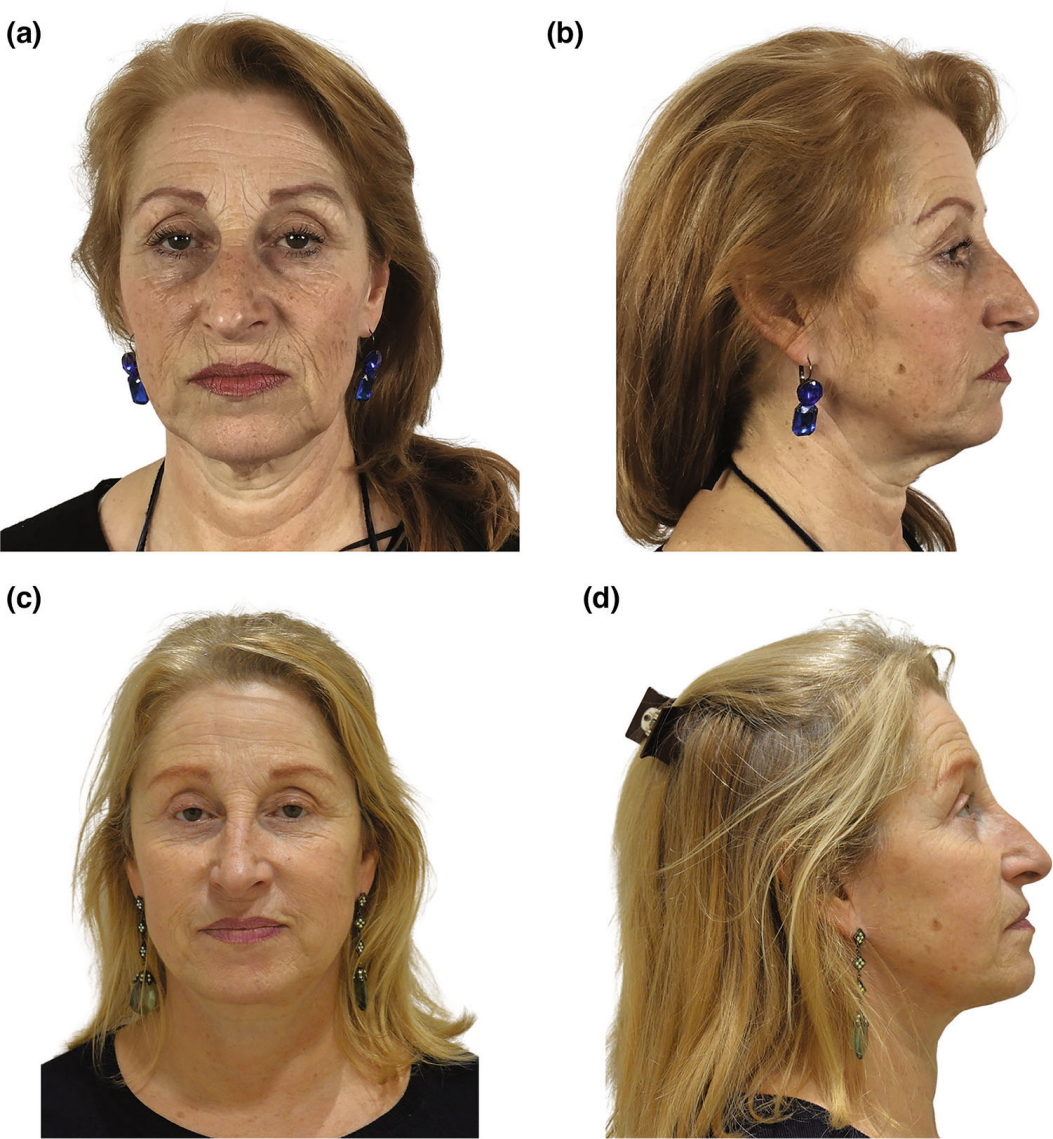
Frontal and lateral photographs of a 56-year-old woman who presented with midline bands, jowls, and skin laxity. She underwent a face and neck-lift along with SMAS flap repositioning, total platysma transection and nanofat injections of the perioral wrinkles. Her preoperative (**a**, **b**) and 6 years postoperative (**c**, **d**) photographs are shown. Improvements in contour of mandible and platysmal bands reduction are shown

**Fig. 5:**
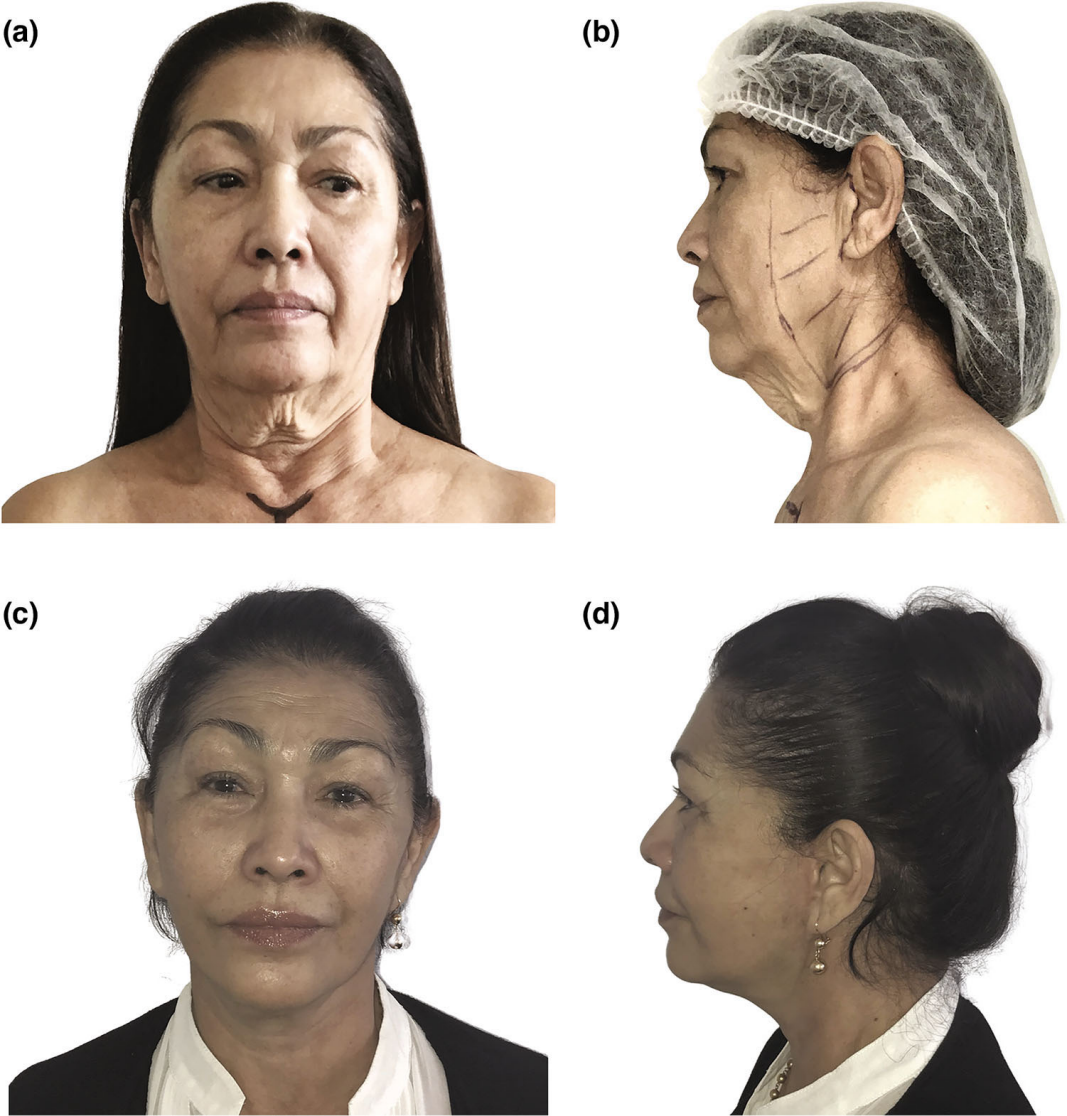
Frontal and lateral photographs of a 67-year-old woman who presented with midline bands, jowls, and a very pronounced skin laxity in the neck region. She underwent a face and neck-lift, SMAS flap repositioning, total platysma transection, neck liposuction. Her preoperative (**a**, **b**) and 5 years postoperative (**c**, **d**) photographs are shown. Improvements in contour of neck mandible and mid-face are shown

**Fig. 6:**
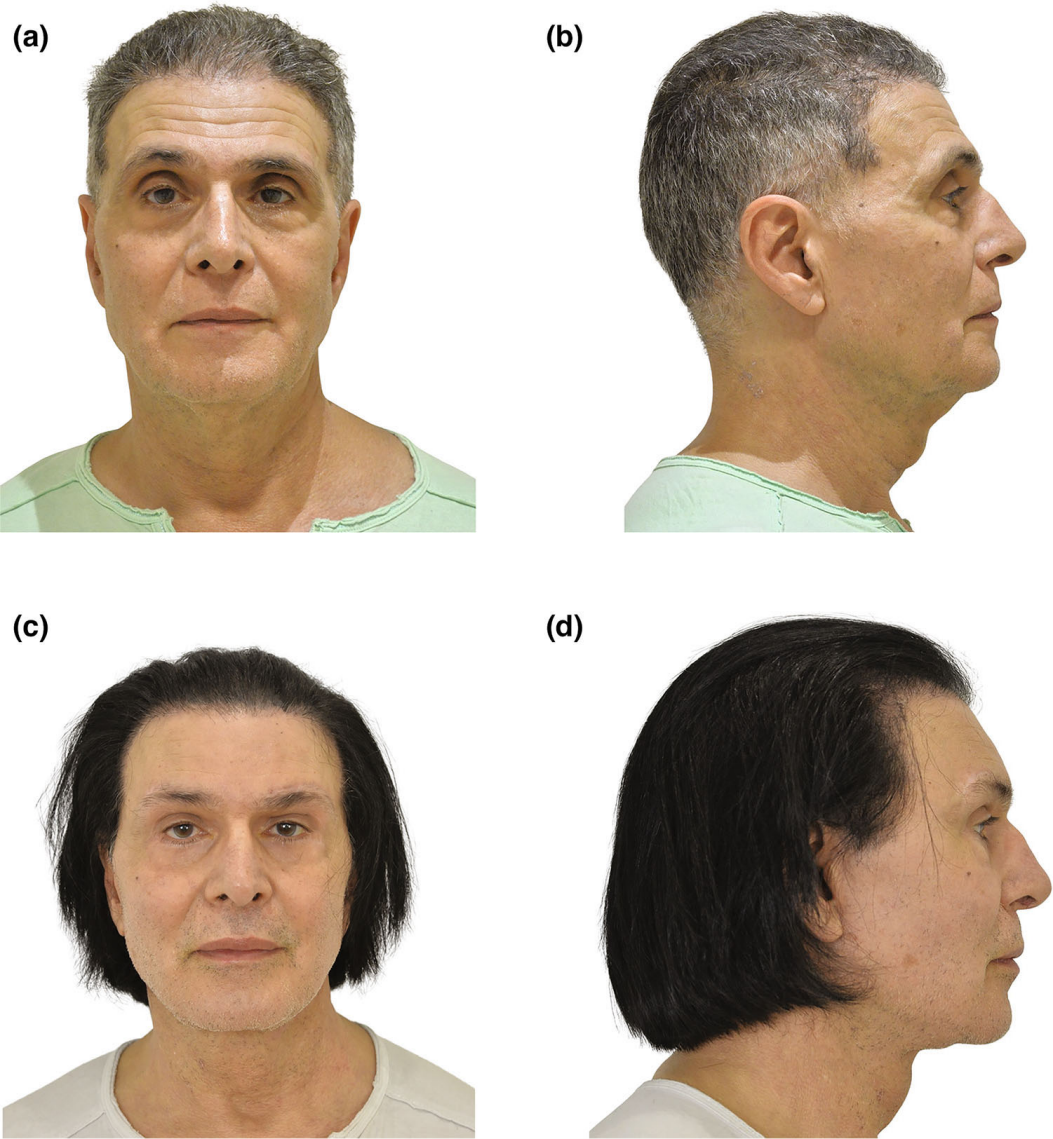
Frontal and lateral photographs of a 63-year-old man who presented with midline bands, jowls, and moderate skin laxity. He underwent a face and neck-lift along with SMAS flap repositioning, total platysma transection, direct brow lift, upper and lower blepharoplasty. His preoperative (**a**, **b**) and 6 years postoperative (**c**, **d**) photographs are shown. Improvements in contour of mandible and neck are shown

**Fig. 7:**
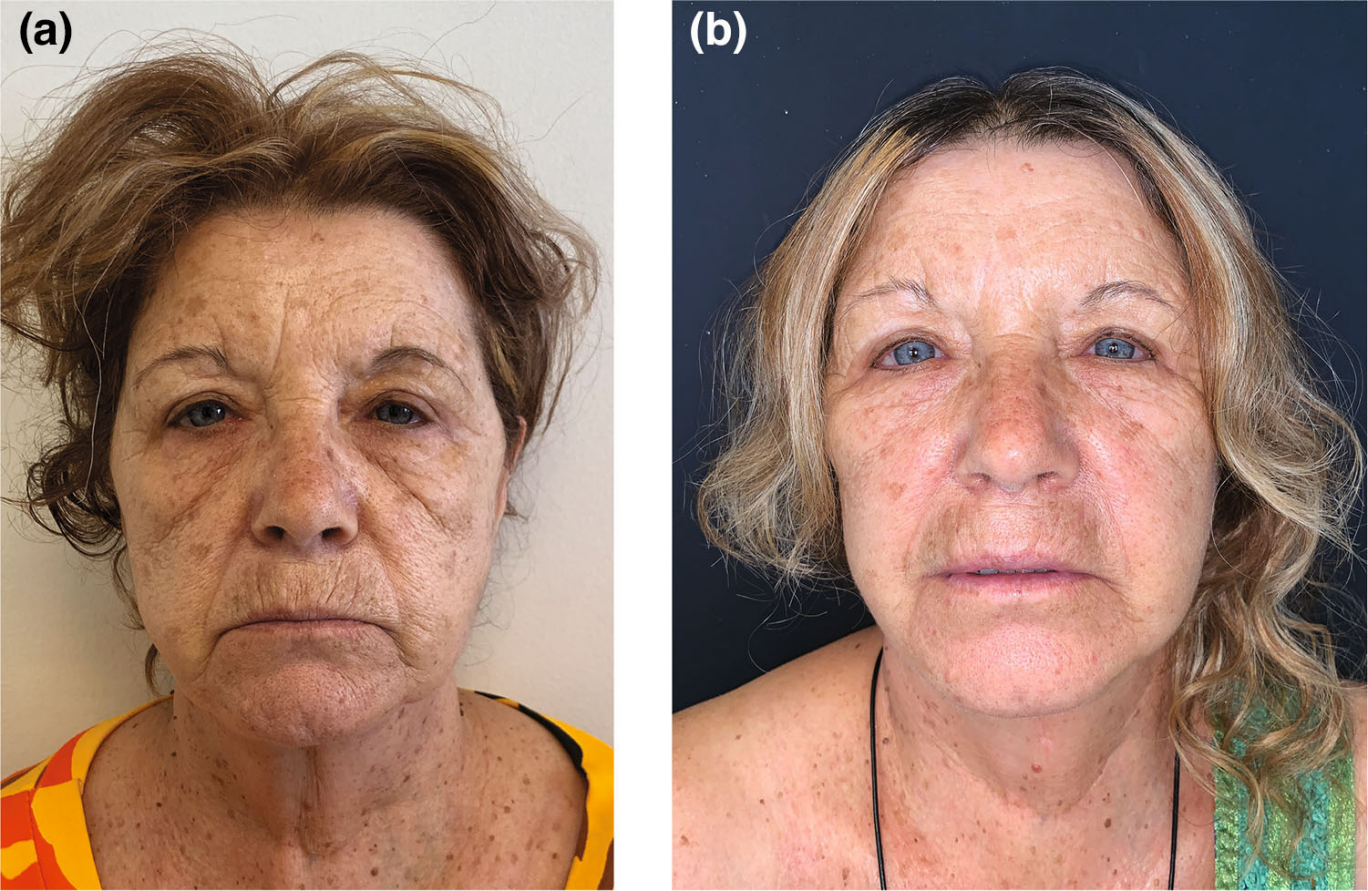
Pre-operative aspect of a 65-year-old woman. She underwent a face and neck-lift along with SMAS flap repositioning, total platysma transection and nanofat injections of the perioral wrinkles and of the malar region. Her preoperative (**a**) and 5 years postoperative (**b**) photographs are shown. Post-operative platysmal bands reduction can be appreciated

**Fig. 8:**
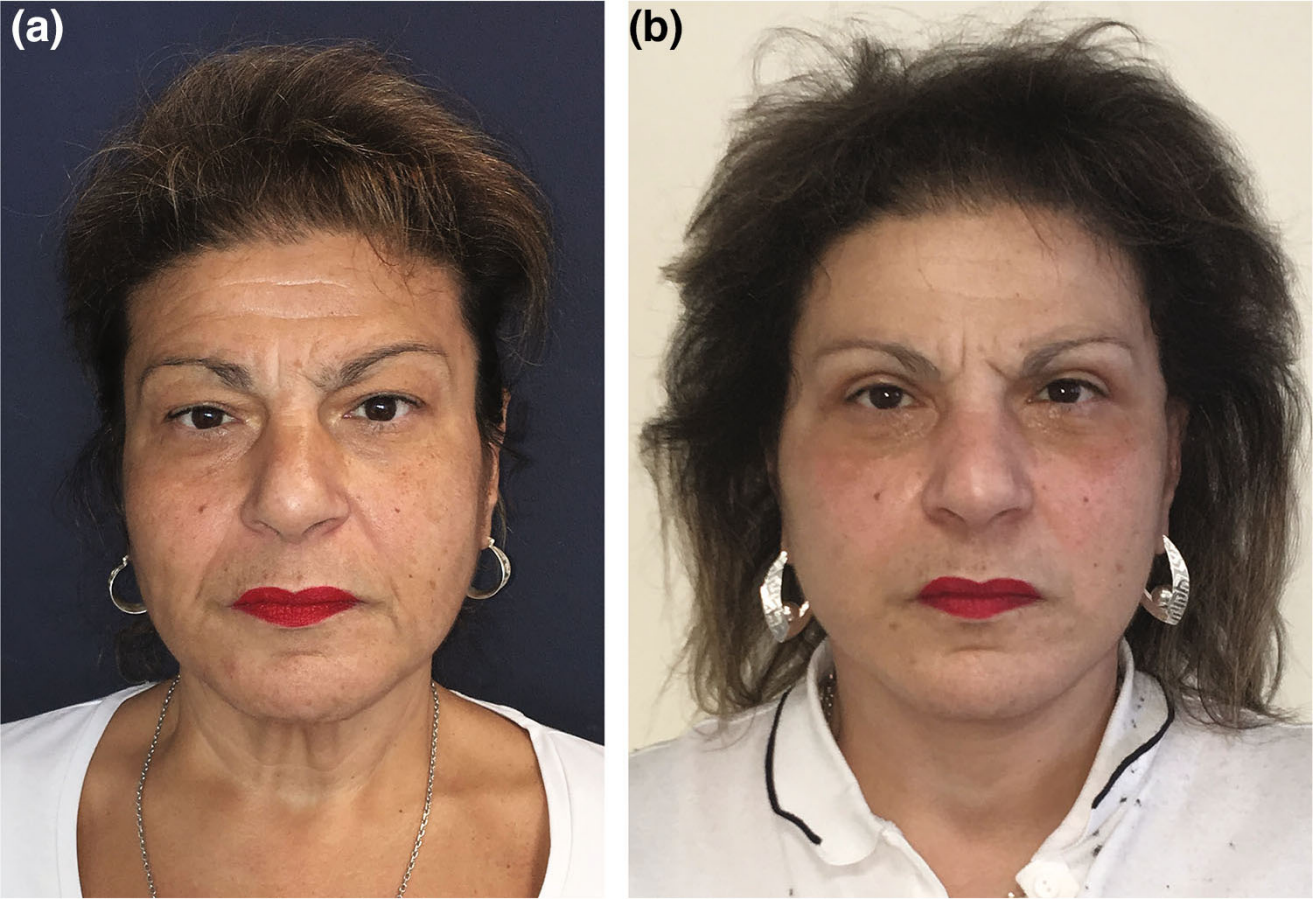
Initial appearance of a 63-year-old woman displaying prominent midline bands prior to treatment. The patient underwent a face and neck-lift procedure involving complete platysma transection. Depicted are her preoperative (**a**) and postoperative at 6 years (**b**) photographs. Evident in the images is the reduction of post-operative platysmal bands

Our clinical study comprised 80 patients who underwent surgery between 2013 and 2016. All the procedures that have been performed are summarized in Table 2. We conducted simultaneous nanofat injections for perioral rhytides in five patients and for the malar area in three patients during the face and neck-lift procedure. In all instances, the fat was harvested from the inner part of the patients’ thighs. Upper and lower blepharoplasty was performed in 25 patients. The mean operative time was 312 ± 51.7 minutes. Patients mean age was 57.43 years (+/− 7.45 years). The average follow-up was 6.5 years (5–8 years). The outcomes were satisfactory, with an improvement in the overall appearance of the treated regions (Figs. 4, 5, 6, 7 and 8). At 5 years, none of the patients exhibited dissatisfaction due to a recurrence of platysma bands. We observed a low complication rate (2.5%), and all complications were effectively managed and resolved through conservative treatment. Out of the total participants, ten patients had a history of prior smoking. Both pre-operative and post-operative urine cotinine tests were negative, indicating their strong adherence to our smoking cessation recommendations. Among these patients, we observed one case of delayed wound healing in the retro-auricular scar of a woman with a history of heavy smoking. Nevertheless, with diligent daily wound care, complete healing was eventually achieved. We observed a single case of infection in the neck area in a patient one week after surgery, which was successfully treated with antibiotics. We did not observe cases of facial paralysis. The difference between the preoperative, 1- and 5-year postoperative scores assessed by three blind evaluators was statistically significant (*p* < 0.001). Specifically, a statistically significant improvement in platysmal bands was observed, as evidenced by the pre- and post-operative scores on the platysmal bands subscale (Table 3).

**Table 2:** Patient characteristics

*Demographic data*	
Age, mean and SD	57.43(± 7.45)
BMI, mean, Kg/m^2^ (SD)	23.43(± 5.62)
*Gender, percentage*	
Men	5(6.2)
Women	75(93.7)
Smokers*, percentage	10(12.5)
*Surgical data*	
Number of procedures	80
Mean operative time, minutes (DS)	312(± 51.7)
Mean follow-up, months (DS)	78.7(± 14.1)
*Surgical procedures*	
Submental lipectomy (%)	60(75)
Total platysmal transection (%)	80(100)
Lateral platysmaplasty (%)	80(100)
Medial platysmaplasty (%)	80(100)
Digastric corset (%)	45(56.2)
Submandibular gland resection (%)	5(6.2)
Nanofat injections (%)	8(10)
Upper and lower blepharoplasty (%)	25(31.25)
Direct brow lift (%)	3(3.75)
*Complication rate (complications conservatively treated)*	2(2.5)
Delayed wound healing (%)	1(1.25)
Infection in the neck area (%)	1(1.25)

*Smokers are identified based on their history of smoking up to one month before the surgery.

**Table 3:** Clinical assessment. To investigate the efficacy of the procedure, the Face- and Neck-Lift Objective Photo-Numerical Assessment Scale was used. Graders were asked to rate pre-, 1 and 5 years post-operative photographs. The mean scores obtained were compared using a paired *T* test. A *p* value < 0.05 was considered significant. Satisfactory results were observed in all cases with significant differences between the mean scores (MS) obtained before one and five years postoperatively. PBS: Platysmal bands subscale

	Pre-operative MS	One-year post-operative MS	Five years post-operative MS	*p* value
Grader 1	29.41 ± 3.21	18.18 ± 3.11	19.11 ± 2.51	< 0.00001
PBS	2.74 ± 0.31	1.15 ± 0.11	1.16 ± 0.12	< 0.0001
Grader 2	31.61 ± 2.32	17.41 ± 2.34	18.31 ± 2.51	< 0.00001
PBS	2.83 ± 0.29	1.10 ± 0.13	1.10 ± 0.65	< 0.0001
Grader 3	29.25 ± 1.93	15.59 ± 1.89	16.72 ± 2.13	< 0.00001
PBS	2.81 ± 0.31	1.14 ± 0.11	1.15 ± 0.12	< 0.0001

## Discussion

Our team has gradually developed an effective technique to address the multiple clinical consequences of facial and cervical aging. Platysmaplasty and SMAS repositioning are of paramount importance as the platysma muscle covers completely the anterolateral cervical region and the lower third of the face [21, 22] in continuity with the SMAS. During the aging process, the platysma muscle shrinks and becomes significantly shorter in the midline and submental region. This process causes the appearance of platysmal bands, which are first visible in motion and eventually even at rest. The loss of the cervicofacial angle can be corrected by lipectomy/liposuction [23–25] in cases caused by adipose infiltration and platysmal corset and platysmal lateral hanging [26, 27]. To some extent, excess skin in the cervical region can be treated without excision. Following cervical lipectomy and the medial platysma corset procedure, the fixation of platysma to the hyoid bone may lead to the resorption of some skin pseudoexcess [28–41]. The total horizontal division of the platysma increases the long-term stability of facelift results and reduces the recurrence rate of platysma bands, as it interrupts muscle and nerve continuity.

The platysmal myotomy performed below the lower border of the submandibular gland offers a dual advantage: preservation of the marginal mandibular branch of the facial nerve (whose lower safe limit is located 4 cm (1.57 inches) below the inferior mandibular border) and suspension of the superior platysma flap, acting as a hammock for the gland and other submandibular tissues. A transection too high would result in the loss of support for part of the submandibular tissue by the platysma.

Since the advent of facelift techniques in the mid-1970s, platysma transection has been a widely employed method for addressing hypertrophic platysma bands in the submental and anterior neck areas [40]. Typically, the platysma has been accessed from its subcutaneous surface following superficial skin undermining, often combined with subcutaneous lipectomy. When the platysma is incised just cephalad to the thyroid cartilage, the incised edges naturally separate by 2–3 cm, effectively elongating the muscle and reducing the contracting band [40–43].

Pelle Ceravolo recently presented a series of 150 patients treated with cervicofacial lift and full section of platysma for the correction of platysmal bands [30]. Despite initial good results (3 months post-op), 35.4% of his patients presented with recurrence of the platysmal bands one year after the operation. Additionally, the author demonstrated increased effectiveness when pulling the SMAS significantly laterally, aligning with our findings.

We believe that partial recurrence of platysmal bands may occur when dissection and myotomy do not involve the inferior cervical area, as the platysma muscle remains innervated by the cervical plexus in this region. Occasionally, we were convinced that we had carried out a complete full-thickness transection of the muscle. However, upon subsequent examination with magnification glasses or an endoscopic device, we found that we had spared some fibers. In these cases, we promptly completed the platysmal myotomy. It is likely that the healing properties of this muscle are very high due to its innervation, and just a few intact fibers are sufficient to cause recurrence.

We were intrigued by the study conducted by Narasimhan et al. [31] in which the authors reviewed their secondary neck-lift procedures. They achieved excellent results using a platysma midline suture and the lateral platysmal window technique, without resorting to complete muscle transection. We concur that for certain cases with mild platysmal bands, the platysmal window technique, as described by Narasimhan et al., could be a viable and effective option. The key elements of our procedure include a full-thickness section of the platysma muscle, significant undermining of the cervical skin from the muscle, creating a low horizontal section to facilitate a ‘hammock’ effect, and maintaining an interval of at least 3 cm (1.2 inches) between the two edges of the transection to prevent recurrences. We hypothesize that an incomplete section of the platysma allows its rich nervous anastomotic network to sustain some muscle activity, leading to self-rehabilitation. Conversely, with a complete muscle section and significant distance between its distal and proximal edges, a lower recurrence of platysmal bands is expected.

Owsley [41] introduced a technique involving high anterior platysma transection of muscle bands at the intended location of the cervicomental angle and at one or more caudal levels along the band, dependent on its length. The width of the primary transection usually extended laterally from the midline for 2–3 cm. A second level of transection, and in rare cases a third, was positioned 3–4 cm below the initial muscle incision for longer or unusually active bands. However, whether he executed a total muscle transection from the medial to the lateral part of the muscle remains uncertain.

We attribute the longer-lasting results achieved with our technique to what we consider essential steps that we systematically follow:

1. Once the platysma is dissected from the skin, we identify both its anterior and posterior edges. Following this, we meticulously undermine the muscle from one edge to the other, starting from the inferior mandibular border and extending downwards to a point located at least 3 cm below the upper border of the thyroid cartilage.
2. We perform a complete transection of the platysma from its medial to its lateral aspect. Using Trepsat scissors, we separate the transection margins of the platysma, creating a gap of no less than 3 cm.
3. Furthermore, after the platysma transection, we systematically inspect the area using magnification glasses and an endoscopic device when necessary. In instances where we identify inadvertently spared fibers, we promptly proceed to complete the platysmal myotomy.
4. Medial platysmaplasty is consistently performed as part of our approach and the platysma is always attached through an anchoring suture to the hyoid bone.

This procedure is challenging and time-consuming for non-experienced surgeons. A high learning curve is needed. Only a precise and perfectly mastered technique can lead to satisfactory results.

However, while we are satisfied with our results, we firmly believe that further studies on this topic, with a larger number of patients, are needed to validate the effectiveness of our method.

### Supplementary Information

Below is the link to the electronic supplementary material.

Supplementary file1 (MOV 54250 KB)
