# Correction: Facelift: Assessment of Total Platysma Muscle Transection to Prevent the Recurrence of Platysmal Bands

**DOI:** 10.1007/s00266-024-04179-8

**Published:** 2024-06-21

**Authors:** Jean-Paul Meningaud, Rosita Pensato, Virginie Pineau, Luca D’Andrea, Chiara Pizza, Edoardo Coiante, Barbara Hersant, Simone La Padula

**Affiliations:** 1 Department of Plastic, Reconstructive and Maxillofacial Surgery, Henri Mondor Hospital, University Paris XII, 1 Rue Gustave Eiffel, 94000 Créteil, France; 2 Department of Plastic and Reconstructive Surgery, Università degli Studi di Napoli Federico II, Via Pansini 5, 80131 Naples, Italy; 3 Paris, France

**Correction to: Aesth Plast Surg (2024) 48:122–133** 10.1007/s00266-023-03664-w

The authors wish to replace Fig. 1 because it belongs to another author and they did not have permission to use it at the time of publication. Figure 1 has been replaced with the original photo taken by Professor Simone La Padula during the anatomical study cited in the article, conducted by Professor La Padula in Paris.

**Fig. 1:**
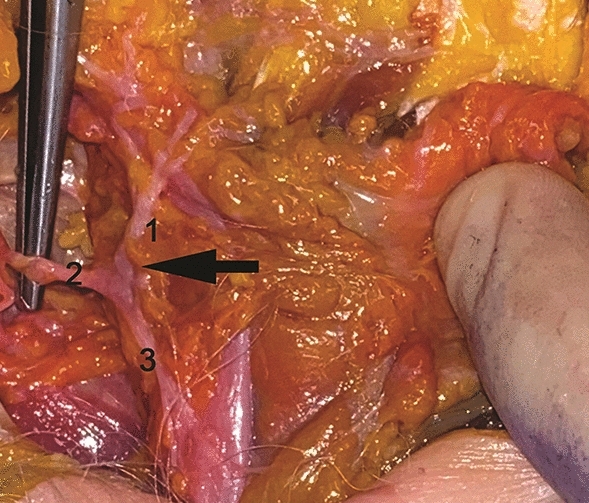
Anatomical findings in fresh cadaver dissections. Ten consecutive cadaveric face and neck dissection (20 hemi-necks and faces) of the superficial musculo-aponeurotic system (SMAS) and platysma areas were carried out to study the innervation of the platysma muscle. Dissections were performed using a facelift approach. The skin and the SMAS were raised very cautiously to reveal the underlying nerves. Innervation of the platysma muscle is shown: The platysma muscle is innervated by the cervical branch of the facial nerve that is connected to the nerves arising from the cervical plexus. A constant anastomotic system between the cervical branch of the facial nerve and the branches of the cervical plexus may be responsible of the platysmal bands recurrence after a facelift procedure, when a complete section of the platysma is not carried out. 1. Marginal mandibular branch of the facial nerve. 2. Cervical branches of the facial nerve. 3. Branches of the cervical plexus. Arrow: Anastomoses between the cervical branch of the facial nerve and branches of the cervical plexus

The authors would also like to add two reference citations to the original article that were previously omitted:

42. Ruiz R, Hersant B, La Padula S, Meningaud JP (2018) Facelifts: Improving the long-term outcomes of lower face and neck rejuvenation surgery: the lower face and neck rejuvenation combined method. J Craniomaxillofac Surg 46(4):697–704. PMID: 29545030.

43. La Padula S, Mernier T, Larcher Q, Pizza C, D’Andrea F, Pensato R, Meningaud JP, Hersant B (2023) Superomedial-posterior pedicle-based reduction mammaplasty: evaluation of effectiveness and BREAST-Q outcomes of a rapid and safer technique. Aesthetic Plast Surg. Epub ahead of print. PMID: 37783863.

The original article has been corrected.

